# Autoimmune Regulator (AIRE) Deficiency Does Not Affect Atherosclerosis and CD4 T Cell Immune Tolerance to Apolipoprotein B

**DOI:** 10.3389/fcvm.2021.812769

**Published:** 2022-01-13

**Authors:** Felix Sebastian Nettersheim, Simon Braumann, Kouji Kobiyama, Marco Orecchioni, Melanie Vassallo, Jacqueline Miller, Amal Ali, Payel Roy, Ryosuke Saigusa, Dennis Wolf, Klaus Ley, Holger Winkels

**Affiliations:** ^1^Department of Cardiology, University Hospital Cologne, Cologne, Germany; ^2^Center for Molecular Medicine Cologne (CMMC), University of Cologne, Cologne, Germany; ^3^La Jolla Institute for Immunology, La Jolla, CA, United States; ^4^Department of Cardiology and Angiology I, University Hospital Freiburg, Freiburg, Germany; ^5^Department of Bioengineering, University of California, San Diego, San Diego, CA, United States

**Keywords:** autoimmune regulator, thymic selection, immune tolerance, atherosclerosis, adaptive immunity, antigen-specific, T cells, dextramer

## Abstract

Atherosclerosis is a chronic, lipid-driven disease of medium sized arteries which causes myocardial infarction and stroke. Recently, an adaptive immune response against the plaque-associated autoantigen Apolipoprotein B100 (ApoB), the structural protein component of low-density lipoprotein, has been implicated in atherogenesis. In healthy individuals, CD4^+^ T cells responding to ApoB mainly comprised regulatory T cells, which confer immune tolerance and atheroprotection. Mice and patients with atherosclerosis harbor increased numbers of proatherogenic ApoB-reactive T-helper cell subsets. Given the lack of therapies targeting proatherogenic immunity, clarification of the underlying mechanisms is of high clinical relevance. T cells develop in the thymus, where strong autoreactive T cells are eliminated in the process of negative selection. Herein, we investigated whether the transcription factor autoimmune regulator (AIRE), which controls expression of numerous tissue-restricted self-antigens in the thymus, is involved in mediating tolerance to ApoB and whether *Aire* deficiency might contribute to atherogenesis. Mice deficient for *Aire* were crossbred to apolipoprotein E-deficient mice to obtain atherosclerosis-prone *Aire*^−/−^ *Apoe*^−/−^ mice, which were fed a regular chow diet (CD) or western-type diet (WD). CD4^+^ T cells responding to the ApoB peptide p6 were analyzed by flow cytometry. We demonstrate that *Aire* deficiency influences neither generation nor activation of ApoB-reactive T cells and has only minor and overall inconsistent impacts on their phenotype. Furthermore, we show that atherosclerotic plaque size is not affected in *Aire*^−/−^ *Apoe*^−/−^ compared to *Aire*^+/+^ *Apoe*^−/−^, irrespective of diet and gender. In conclusion, our data suggests that AIRE is not involved in regulating thymic expression of ApoB or atherosclerosis. Alternative mechanisms how ApoB-reactive CD4 T cells are selected in the thymus will have to be investigated.

## Introduction

Atherosclerosis is a chronic condition of large- and medium-sized arteries that involves formation of leukocyte- and lipid-rich plaques in the arterial wall (1). Atherogenesis is accompanied and modulated by CD4^+^ T cells responding to plaque-associated autoantigens (2, 3). One of these autoantigens is Apolipoprotein B (ApoB), the core protein of low-density lipoprotein (LDL) and other lipoprotein particles (2). ApoB-reactive CD4^+^ T cells (ApoB^+^ T cells) can be detected in peripheral blood mononuclear cells (PBMCs) and lymph nodes from healthy and atherosclerotic humans and mice, respectively (4, 5). In healthy individuals these cells are mainly regulatory T cells (T_regs_) (4), which confer atheroprotection in mice (6, 7) and predict lower cardiovascular event rates in humans (8). T_regs_ express the transcription factor (TF) forkhead box protein P3 (FOXP3) and maintain peripheral immune tolerance by attenuating pathogenic (auto-)immune responses through multiple mechanisms including secretion of the anti-inflammatory cytokines interleukin-10 (IL-10) and transforming growth factor beta (TGF-β) (9). In atherosclerotic individuals, ApoB^+^ T cells frequently coexpress TFs typical found in pro-atherogenic T helper 1 (T_H_1) or T helper 17 (T_H_17) cells (4).

Central T cell tolerance develops in the thymus, where bone-marrow-derived progenitors undergo a complex maturation and selection process, of which in the end only few single CD4^+^ or CD8^+^ T cell receptor (TCR)-αβ expressing T cells will leave into the periphery (10, 11). TCR gene segment rearrangement catalyzed by recombinase activating gene (RAG) 1 and 2 is stochastic and yields a unique combination of a TCR-α and -β chain. At this stage, so-called double-positive (DP) thymocytes express the TCR co-receptors CD4 and CD8, which aid in binding of the TCR to peptide-loaded major histocompatibility complexes (MHC) I and II, respectively. The newly formed TCR will be functionally tested in the process of positive and negative selection. DP cells with a functional TCR that recognize ubiquitous self-antigens presented on MHC class I or II molecules with sufficient affinity pass this selection process, downregulate either CD8 (when binding to MHC II) or CD4 (when binding to MHC I), and mature into CD4 or CD8 single positive (SP) cells (12). Too strong interactions of DP cells with ubiquitous self-antigens lead to apoptosis (cortical negative selection) (12). Surviving SP cells migrate into the thymic medulla, where they undergo negative selection (12). Only SP cells with low affinity to tissue-restricted antigens (TRAs; antigens only expressed by one or a few anatomical sites), which are mainly presented by medullary thymic epithelial cells (mTECs) and to a lesser extent by other cell types such as thymic B cells or dendritic cells (DCs), survive this process and mature into effector T cells (T_eff_ cells) (12). SP cells with high affinity to TRAs undergo apoptosis and a subgroup of CD4 SP cells with intermediate affinity develop into invariant natural killer T or natural regulatory T_regs_ (nT_regs_) which are also termed thymic T_regs_ (tT_regs_) (13, 14). The expression of many - but not all - peripheral tissue genes in mTECS is regulated by the two transcription factors AIRE and FEZF2 (15–17). Mutations in the *AIRE* gene cause a rare autoimmune disorder in humans, which is termed autoimmune polyglandular syndrome type I (APS 1) or autoimmune polyendocrinopathy-candidiasis-ectodermal dystrophy (APECED) and involves chronic mucocutaneous candidiasis as well as dysfunction of the adrenal, parathyroid and other endocrine glands (18, 19). Mice deficient for AIRE show increased numbers of antigen-specific effector T cells and develop multi-organ autoimmunity (15, 20–22). Yet, it is unclear whether AIRE controls the expression of ApoB and the autoimmune CD4^+^ T cell response in atherosclerosis. ApoB expression is high in mature mTECs, but almost absent in immature mTECs, cortical TECs (cTECs), thymic DCs, and macrophages (23–25). Whereas Sansom and colleagues found highly increased ApoB mRNA levels in mature AIRE-positive mTECs compared to AIRE-negative mTECs or mTECS from *Aire*-deficient mice (Supplementary Figure 1) (25), others reported similar ApoB expression in mature mTECs isolated from *Aire*-deficient mice and wildtype controls (15, 24).

Herein we crossed *Aire-* and *apolipoprotein E* (*Apoe*)-deficient (*Aire*^−/−^ *Apoe*^−/−^) mice to test whether lack of AIRE would increase peripheral ApoB-reactive effector T cell numbers and functions and increase atherosclerosis. We demonstrate that AIRE-deficiency does neither affect generation of ApoB^+^ T cells nor their phenotypes and has no impact on atherogenesis, pointing toward a dominant role of an alternative central or peripheral mechanism in enabling immune tolerance to ApoB.

## Methods

### Mice

Autoimmune regulator–deficient (*Aire*^−/−^) mice on C57BL/6J background were purchased from Jackson Laboratories (cat. #004743 Bar Harbor, ME) and crossbred with apolipoprotein E-deficient (*Apoe*^−/−^) mice to obtain *Aire*^−/−^*Apoe*^−/−^ mice. Mice were housed in a specific pathogen–free environment and fed chow diet (CD) until 10 weeks of age. At 8 weeks of age, mice were either fed CD or western-type diet (WD), adjusted calories diet with 42% from fat (Harlan Labs Cat #: TD.88137, CA, USA) and remained on CD or WD for 12 weeks until organ collection. All animal experiments were conducted in accordance with the institutional guideline for the La Jolla Institute for Immunology animal facility.

### Atherosclerosis Quantification

The whole aorta (thoracic and abdominal) was excised, cleaned *in situ*, and pinned out after paraformaldehyde incubation at room temperature for at least 2 h. Atherosclerotic lesions were visualized by Sudan-IV staining and quantified as the percentage Sudan-IV-positive area of the size of the whole aorta. Quantification was performed using ImagePro software (Media Cybernetics, Rockville, MD, USA).

### Flow Cytometry

Cell suspensions were prepared from thymus, blood, spleen, or pooled lymph nodes (axillary, cervical, inguinal, para-aortic, mesenteric) and incubated with fluorochrome-coupled antibodies against the indicated antigens for 20 min at RT in RPMI-1640 containing 10% rat serum and 10 μg/ml antiCD16/CD32 antibodies to block unspecific Fc-receptor interactions. Cells were washed in PBS and fixed in 2% Paraformaldehyde (PFA) for 10 min. If not otherwise indicated, T-helper cells were identified as CD4^+^ TCR-β^+^ Lin^−^ L/D (live/dead dye, Tonbo Biosciences, San Diego, Ca, USA)^−^. Lin contained antibodies against CD11b, F4/80, CD19, B220, CD11c, Nk1.1, TER-119, and CD8. Transcription factors were stained with a permeabilization/fixation protocol according to the manufacture's recommendations (Thermo Fisher Scientific, Waltham, MA, USA). T_regs_ were identified as CD4^+^ Foxp3^+^ CD25^+^, T_H_1 cells were identified as CD4^+^ Foxp3^−^ T-bet^+^, T_H_17 cells were identified as CD4^+^ Foxp3^−^ RORγt^+^, T_H_2 cells were identified as CD4^+^ Foxp3^−^ T-bet^−^ RORγt^−^ GATA3^+^, and T follicular helper (T_FH_) cells were identified as CD4^+^ T-bet^−^ RORγt^−^ Bcl-6^+^. T-effector memory subsets were identified as T_EM_ (CD44^+^ CD62L^−^), T-central memory cells as T_CM_ (CD44^+^, CD62L^+^), and naïve T cells as T_naï*ve*_ (CD44^−^ CD62L^+^). To stain intracellular cytokines (IL-17A, IL-12p40, TNF-α, IFN-γ, IL-10, and IL-4), single cell suspensions were prepared from pooled lymph nodes and stained with anti-CD45 (30-F11: Thermo Fisher Scientific), anti-CD4 (RM4–5: Biolegend; San Diego, CA), and anti-TCRβ (H57–597: Thermo Fisher Scientific) antibodies. Subsequently, the cells were stimulated with cell stimulation cocktail (Thermo Fisher Scientific) and monensin (Thermo Fisher Scientific) for 5 h. Dead cells were identified by staining with Ghost Dye UV450 (Tonbo Biosciences) and IC Fixation Buffer (Thermo Fisher Scientific) was used for fixation and permeabilization. Samples were acquired with a FACS LSR-II or FACS Fortessa (BD Biosciences, San Diego, CA, USA). If not stated otherwise, all anti-mouse antibodies were purchased from Biolegend (San Diego, CA, USA) and used in a final dilution of 1:50 (cytokines/transcription factors/cytoplasmatic proteins) and 1:200 (extracellular markers). Data were analyzed with FlowJo software (Treestar, San Diego, CA, USA).

### ApoB:MHC-II Multimers

Biotinylated ApoB:MHC monomers, in which the peptide ApoB_978−993_ was fused to I-A^b^ (the MHC-II allele expressed by C57BL/6J mice), were coupled to streptavidin-phycoerythrin or streptavidin-allophycocyanin labeled dextran backbones by Immudex (Copenhagen, Denmark) for the identification of ApoB-reactive CD4^+^ T cells. ApoB:MHC dextramers were generated as described previously (26) and had the following specifications: ApoB:MHC dextramer-PE carried ~20 ApoB:MHC monomers and ~4 PE fluorochromes per dextran, while ApoB:MHC dextramer-APC carried ~12 7 ApoB:MHC monomers and ~9 APC fluorochromes per dextran.

### Peptide:MHC Multimer Staining

Cell suspensions were prepared from pooled lymph nodes. Before incubation with multimers, CD4^+^ T cells were enriched by a negative magnetic bead separation with biotinylated anti-CD11b, -CD11c, -TER119, -CD8, -F4/80, -Nk1.1, -B220, -CD19 antibodies (Tonbo Biosciences) and streptavidin-coupled magnetic microbeads (MagniSort, Thermo Fisher Scientific). Enriched CD4^+^ T cells were > 90% pure, based on surface expression of TCR-β and CD4 as measured in flow cytometry. ApoB:MHC-streptavidin-PE and ApoB:MHC-streptavidin-APC dextramers were incubated for 1 h at room temperature in the dark in RPMI supplemented with 10% rat serum and 10 μg/ml anti-CD16/32 (Tonbo Biosciences). The final concentration for ApoB-MHC was 2.5 nM. Cells were subsequently stained for the indicated surface markers and live-dead dye (eF450 viability dye, Thermo Fisher Scientific). Dextramer staining was validated by a fluorescence-minus-two (FMT) control (APC and PE; Supplementary Figure 2). Non-T cells were identified by fluorochrome-labeled antibodies against CD11b, CD11c, F4/80, CD19, CD8, B220 (Lineage, all from Biolegend,). For extracellular staining, cell suspensions were fixed in 2% PFA. As indicated, intracellular staining for transcription factors was performed according to the manufacturer's instructions (Thermo Fisher Scientific). Data were analyzed with FlowJo software (Treestar). For calculation of the absolute numbers of ApoB^+^ T cells, leukocyte numbers of enriched CD4^+^ lymph node T cells were quantified (Hemavet, DrewScientific, Miami Lakes, USA) before multimer staining.

### Statistics

Data are presented as mean ± SD. Differences between the groups were evaluated using one-way repeated measures analysis of variance (ANOVA) with a *post-hoc* Tukey's test. *P*-values < 0.05 were considered statistically significant. All statistical analyses were performed using GraphPad Prism 8.4.0 (GraphPad Software, San Diego, CA, USA).

## Results

### AIRE Deficiency Has No Relevant Impact on Thymopoiesis in *Apoe*^–/–^ Mice

**Figure 1:**
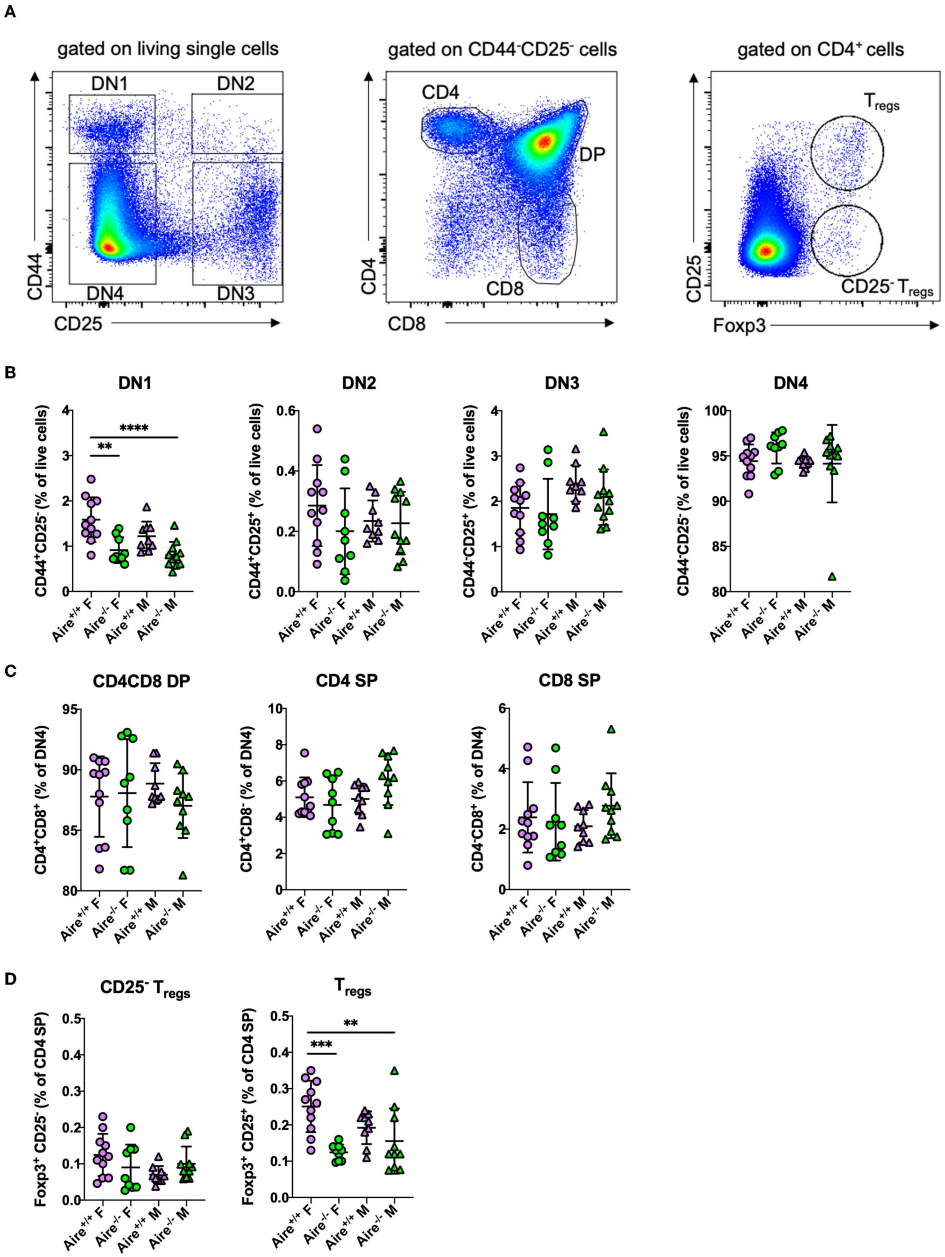
Thymopoiesis is largely unaffected by AIRE deficiency in male and female *Apoe*^−/−^ mice. **(A)** Gating scheme for flow-cytometric detection of maturating thymocytes: Double negative (DN) 1 (CD44^+^CD25^−^), DN2 (CD44^+^CD25^+^), DN3 (CD44^−^CD25^+^), and DN4 (CD44^−^CD25^−^) cells were identified by expression of the surface markers CD44 and CD25. CD4 and CD8 single positive (SP) or double positive (DP) cells were identified as CD44^−^CD25^−^ cells with expression of CD4 and/or CD8. Regulatory T cells were identified as Foxp3^+^ CD25^+^ CD4^+^ cells and CD25^−^ T_reg_ precursors were identified as Foxp3^+^ CD25^−^ CD4^+^ cells. **(B,C)** Quantification of different stages in thymic development in 20-week-old male and female *Aire*^−/−^*Apoe*^−/−^ and *Aire*^−+/+^*Apoe*^−/−^ fed western-type diet for 12 weeks. **(D)** Quantification of thymic CD25^−^ and CD25^+^ regulatory T_regs_. **(B–D)** *n* = 9–11 per group. Data are expressed as mean ± SD. Statistical significance was determined by one-way ANOVA with Tukey's multiple comparisons test. **p < 0.01, ***p < 0.001, ****p < 0.0001.

Despite its central role in mediating negative selection of CD4^+^ T cells, AIRE does not globally influence frequencies of maturing thymocytes (15, 22). To determine whether AIRE impacts thymopoiesis in *Apoe*^−/−^ mice, we quantified different stages of T cell development by flow cytometry (Figure 1A). The earliest developing thymocytes do neither express CD4 nor CD8 and are thus called double negative (DN) cells. According to expression of the surface markers CD44 and CD25, DN cells are subdivided into four stages of maturity: DN1 (CD44^+^CD25^−^), DN2 (CD44^+^CD25^+^), DN3 (CD44^−^CD25^+^), and DN4 (CD44^−^CD25^−^) cells. The DN4 population (DN4) further develops into double positive CD4^+^CD8^+^ cells, which mature into single positive CD4^+^ or CD8^+^ T cells (10). Aire deficiency was associated with a reduction of DN1 cells, but did not affect subsequent developmental stages (Figures 1B,C). We thus confirm Aire does overall not exert relevant influences on thymopoiesis in *Apoe*^−/−^ mice, which is in accord with findings obtained in wildtypes (22). Considering that Aire affects thymocytes at the SP stage, causes and significance of the observed decrease in DN1 cell counts remain elusive and await confirmation in future studies.

Besides inducing elimination of specific autoreactive thymocytes, Aire was implicated in directing these cells into the T_reg_ line (27). Aire-deficient mice were repeatedly reported to harbor less thymic T_regs_ than wildtype controls (28, 29). Thymic T_regs_ classically develop from CD4^+^ T cells through subsequent induction of CD25 and Foxp3 (30). Besides that, an alternative developmental route has been described, in which Foxp3 is expressed before CD25 (30). Frequencies of CD25^−^Foxp3^+^ T_reg_ precursors did not differ between the groups (Figure 1D). We observed a reduced number of CD25^+^ Foxp3^+^ T_regs_ in the thymus of female—but not male^−^*Aire*^−/−^*Apoe*^−/–^ mice (Figure 1D), which is in contrast to observations made in male wildtype mice (29). Future studies will have to address, whether the gender effect might be overshadowed by the atherosclerotic environment.

### Aire-Deficient *Apoe*^–/–^ Mice Do Not Display Broad Immunological Abnormalities

AIRE deficiency did not affect leukocyte composition in the blood, spleen and lymph nodes in male and female *Apoe*^−/−^ mice fed a WD (Supplementary Figure 3). In comparison to male *Aire*^+/+^*Apoe*^−/−^ mice, *Aire*^+/+^*Apoe*^−/−^ females had a modest yet statistically significant increases in CD4^+^, CD8^+^, and γδ T cell counts in lymph nodes, whereas female *Aire*^−/−^*Apoe*^−/−^ mice exhibited higher levels of γδ T cells in the spleen than *Aire*^−/−^*Apoe*^−/−^ males. We did not detect relevant differences in body weight or hematological parameters between *Aire*^+/+^*Apoe*^−/−^ and *Aire*^−/−^*Apoe*^−/−^ mice, (Supplementary Table 1). Aire deficiency was associated with an increased neutrophil count in female *Apoe*^−/−^ mice exposed to a WD and with an increase in eosinophils and decrease in platelets in CD-fed male *Apoe*^−/−^ mice. Furthermore, WD-fed males had a lower proportion of monocytes and CD-fed males had higher relative neutrophil and lower relative lymphocyte counts compared to females. Collectively, these data indicates that Aire deficiency does not induce broad alterations of innate and adaptive immunity.

### Frequency and Activation of ApoB^+^ T Cells Are Not Influenced by Aire

**Figure 2:**
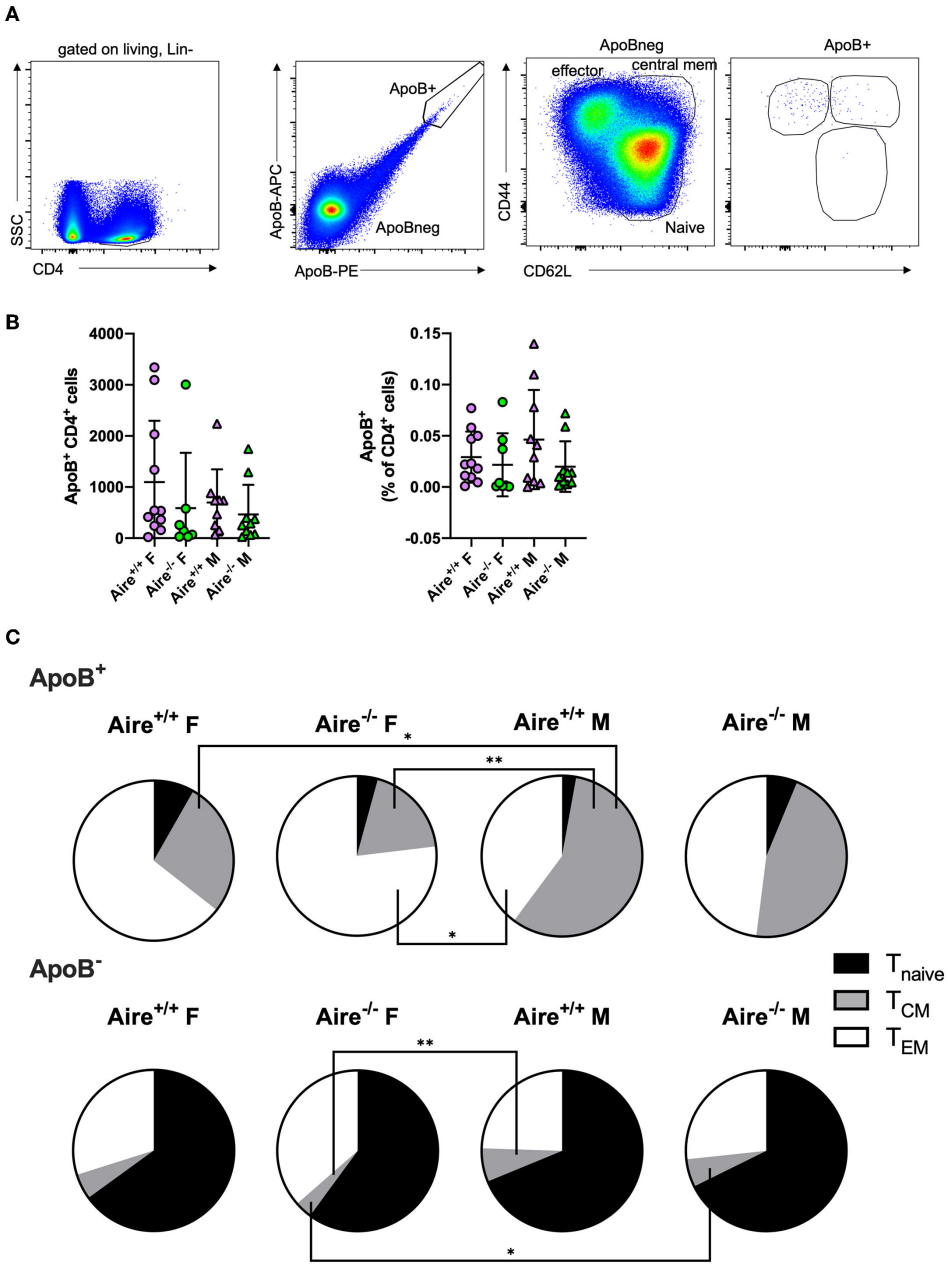
Aire deficiency does not affect generation and activation of ApoB-reactive CD4^+^ T cells. **(A)** Gating scheme for identification of ApoB^+^ and ApoB^−^ CD4^+^ T effector memory (T_EM_, CD44^+^CD62L^−^), T central memory (T_CM_, CD44^+^CD62L^+^) and naïve T cells (T_nave_, CD44^−^CD62L^+^) by flow-cytometry. **(B)** Absolute counts (left) and frequency (% of CD4^+^ T cells, right) of ApoB^+^ T cells isolated from lymph nodes of 20-week-old male and female *Aire*^−/−^*Apoe*^−/−^ and *Aire*^−+/+^*Apoe*^−/−^ fed western-type diet for 12 weeks. Data are presented as mean ± SD. **(C)** Quantification of T_EM_, T_CM_, and T_nave*ve*_ ApoB^+^ and ApoB^−^ T cells. Pie charts show the group means of indicated cell types. **(B,C)** *n* = 7–11 per group. Statistical significance was determined by one-way ANOVA with Tukey's multiple comparisons test. *p < 0.05, **p < 0.01.

We have recently identified ApoB-reactive CD4^+^ T cells in lymph nodes of healthy and atherosclerotic mice (5). Herein, we sought to evaluate whether Aire affects generation and activation of atherosclerosis-relevant ApoB^+^ T cells in *Apoe*^−/−^ mice. We utilized a previously published fluorochrome-labeled multimer (5) consisting of recombinant I-A^b^ (the MHC-II allele expressed by C57Bl/6 mice) complexed with the ApoB peptide p6 to identify ApoB^+^ T cells in lymph nodes by flow cytometry (Figure 2A). We detected ~500–1,000 ApoB^+^ cells (0.02–0.05% of all CD4^+^ T cells) in male and female *Aire*^−/−^ and *Aire*^+/+^ mice, without any apparent differences between the four groups (Figure 2B). To evaluate the impact of *Aire* deficiency on activation of ApoB^+^ and ApoB^−^ T cells, surface expression of CD44 and CD62L in lymph node CD4^+^ T cells was analyzed (Figure 2A). In line with previous data (5), the majority of ApoB^+^ cells consisted of antigen-experienced T-effector memory (T_EM_, CD44^+^CD62L^−^) and T central memory (T_CM_, CD44^+^CD62L^+^) cells, whereas only few naive ApoB^+^ T cells (CD44^−^CD62L^+^) were detectable (Figure 2C). In contrast, ApoB^−^ cells were predominantly antigen-unexperienced. AIRE-deficiency did not change antigen-experienced T_CM_/T_EM_ ApoB^+^ and ApoB^−^ cells (Figure 2C, Supplementary Figures 4A,B). Previous studies have reported conflicting findings regarding the impact of AIRE on CD4^+^ T cell activation: Whereas Anderson et al. observed a near-doubling of CD4^+^ T_EM_ cells in lymph nodes of *Aire*^−/−^ compared to *Aire*^+/+^ mice (15), Ramsey and colleagues did not detect such a difference (20). Here we found that ApoB^−^ T_EM_ cells were 25% of all CD4^+^ T cells, regardless of AIRE expression, and thus twice as high as previously detected by Anderson et al. in *Aire*^−/−^ mice (12%) (15). We therefore suggest that the inflammatory environment in WD-fed *Apoe*^−/–^ mice largely overlayed any potential AIRE-deficiency caused effects on T cell activation. Female Aire-competent mice exhibited a significantly lower frequency of ApoB^+^ T_CM_ cells compared to males. Additionally, AIRE-deficient females had significantly fewer ApoB^−^ T_CM_ cells than male *Aire*^−/−^ mice. However, overall CD44^+^ cells did not differ between males and females, arguing against a relevant gender dependent T cell activation (Supplementary Figure 4C). Collectively, these data indicate that AIRE exerts an insignificant effect on the generation and activation of ApoB^+^ cells in male and female *Apoe*^−/–^ mice.

### Aire Has Minor Impacts on Phenotypes of Antigen-Experienced ApoB^+^ T Cells

**Figure 3:**
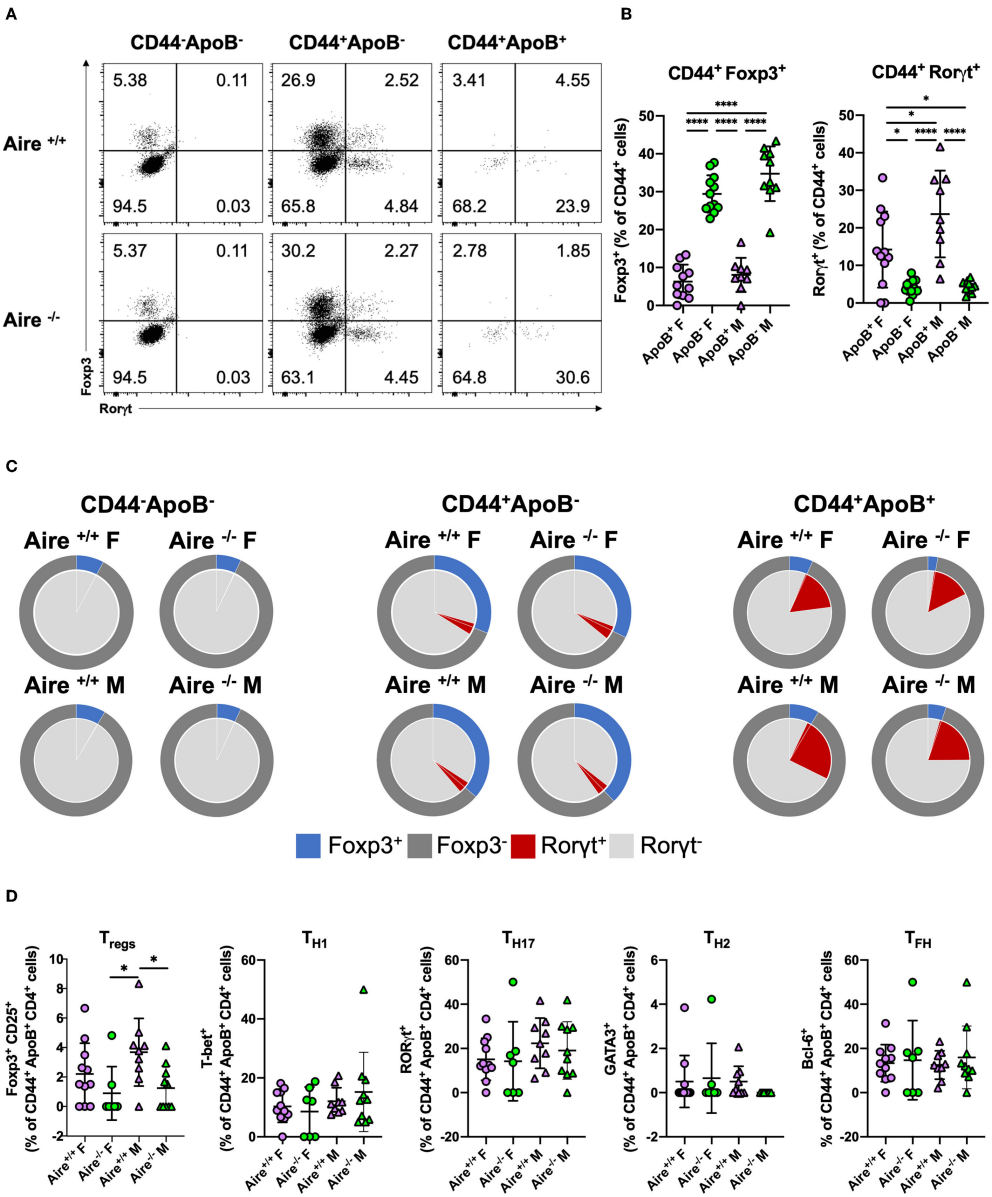
Phenotypes of antigen-experienced ApoB^+^ T cells are marginally influenced by AIRE deficiency. **(A)** Expression of the transcription factors Foxp3 and RORγt in CD44^−^ ApoB^−^, CD44^−^ ApoB^+^, and CD44^+^ ApoB^+^ cells analyzed by flow-cytometry. **(B,C)** Quantification of Foxp3^+^ and RORγt^+^ among CD44^+^ ApoB^+^ and ApoB^−^ cells isolated from lymph nodes of 20-week-old male and female *Aire*^−/−^*Apoe*^−/−^ and *Aire*^−+/+^*Apoe*^−/−^ fed western-type diet for 12 weeks. **(D)** Quantification of CD4 T cell lineage transcription factors T-bet (T_H_1), GATA3 (T_H_2), BCL6 (T_FH_), Foxp3 (T_regs_) and RORγt (T_H_17). **(B,D)** Data are expressed as mean ± SD. **(C)** Pie charts show the group means of indicated cell types. **(B–D)** *n* = 7–11 per group. Statistical significance was determined by one-way ANOVA with Tukey's multiple comparisons test. *p < 0.05, ****p < 0.0001.

We have recently shown that ApoB^+^ T cells acquire a proinflammatory phenotype during atherogenesis. Antigen-experienced (CD44^+^) ApoB^+^ T cells comprised a significantly lower proportion of Foxp3^+^ T_regs_ and more RORγt (the lineage defining TF of T_H_17 cells) expressing cells compared to CD44^+^ApoB^−^ T cells in atherosclerotic mice (5). To evaluate whether AIRE influences the phenotypes of antigen-experienced and -unexperienced ApoB^+^ and ApoB^−^ T cells we quantified the expression of Foxp3, RORγt (Figure 3A) and other lineage-defining TFs in these cells. In line with our previous findings (5), we detected significantly fewer Foxp3^+^ and more RORγt^+^ cells within the pool of CD44^+^ ApoB^+^ cells compared to CD44^+^ ApoB^−^ cells (Figures 3B,C). Foxp3 and RORγt expression in CD44^+^ and CD44^−^ ApoB^−^ T cells was not affected by AIRE deficiency (Figure 3C, Supplementary Figure 5A). Irrespective of AIRE expression, CD44^+^ ApoB^+^ T cells mainly comprised proinflammatory T-bet^+^ T_H_1 cells, RORγt ^+^ T_H_17 cells, and Bcl-6^+^ T_FH_ cells, which all did not express Foxp3 (Figure 3D). In atherosclerotic male and female *Aire*^−/−^ mice, the frequency of CD25^+^ Foxp3^+^ T_regs_ was modestly decreased within CD44^−^ ApoB^−^ cells. AIRE-deficiency did not alter the phenotypes of CD44^+^ ApoB^−^ and CD44^−^ cells (Supplementary Figure 5B). The observed reduction of CD44^+^ ApoB^+^ T_regs_ in atherosclerotic *Aire*^−/−^ mice is in line with impaired thymic generation of T_regs_ in female *Aire*^−/−^ *Apoe*^−/−^ mice (Figure 1D). The consistency and relevance of these gender-specific observations are yet unclear and require confirmation in future studies.

Aire deficiency did not substantially affect expression of hallmark pro- and anti-inflammatory cytokines in activated CD4^+^ T cells (Supplementary Figure 6). Whereas, no significant differences in lineage hallmark cytokines were detected between the groups, CD44^+^ CD4^+^ T cells of female *Aire*^−/−^ mice produced less TNF-α (which is mainly produced by T_H_1 cells) compared to *Aire*^+/+^ mice. Activated CD4^+^ T cells of female *Aire*^+/+^ (and to a lesser extent *Aire*^−/−^) mice expressed higher amounts of IL-4 (typical for T_H_2 cells) compared to males. The role of IL-4 in atherosclerosis is controversial (2) and gender-specific effects have not yet been described.

### Aire Deficiency Does Not Affect Atherogenesis in *Apoe*^–/–^ Mice

**Figure 4:**
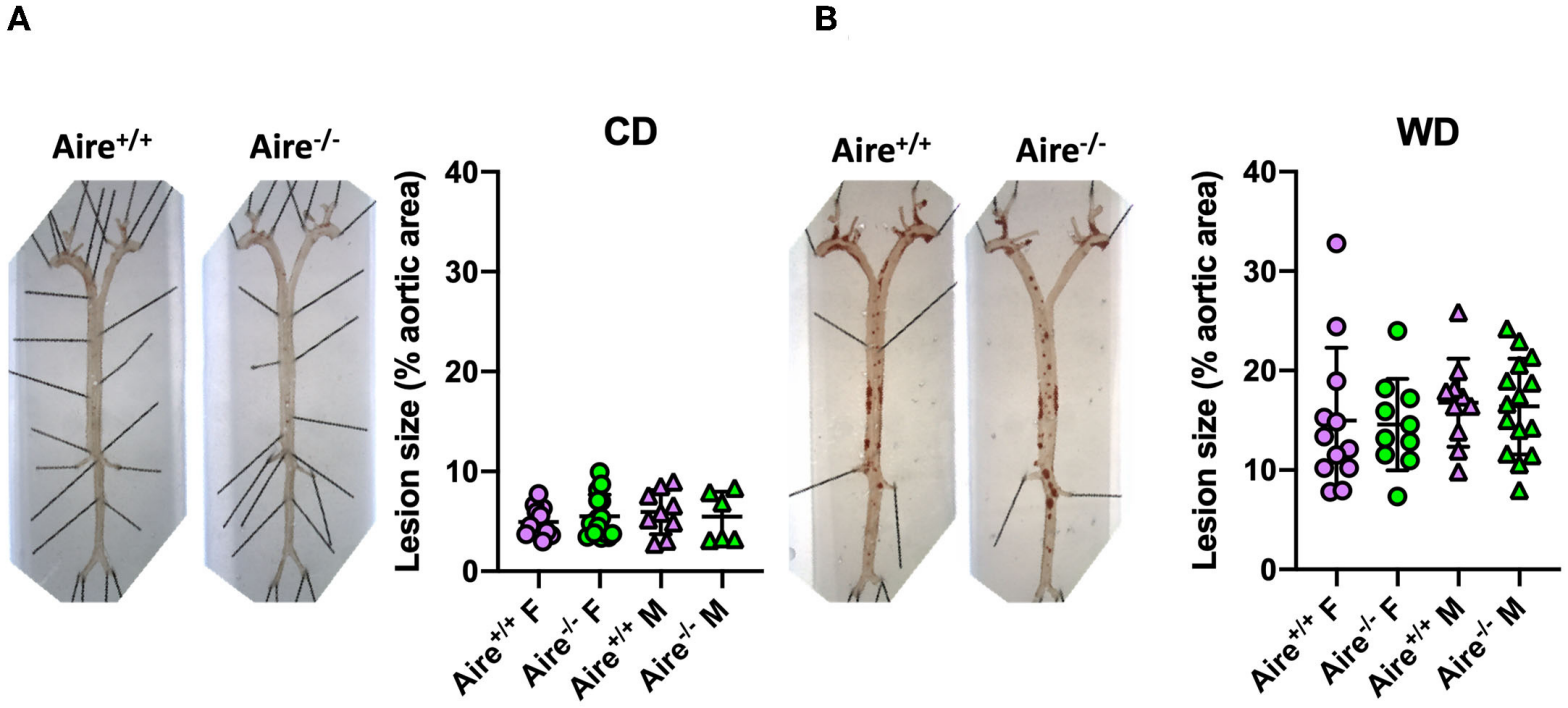
Atherosclerotic lesion size is not influenced by Aire deficiency. Representative images quantification of Sudan IV-stained atherosclerotic plaques within the aorta of 20-week-old male and female *Aire*^−/−^*Apoe*^−/−^ and *Aire*^−+/+^*Apoe*^−/−^ mice **(A)** fed chow diet (*n* = 6–15 per group) and **(B)** western-type diet *Apoe*^−/−^ mice (*n* = 10–15 per group). Plaque size was calculated as percentage of whole aortic area. Data are expressed as mean ± SD. Statistical significance was determined by one-way ANOVA with Tukey's multiple comparisons test.

To determine whether AIRE deficiency affects atherogenesis, we quantified en face atherosclerotic lesions in the aorta. In line with previous data (31), lesion size in *Apoe*^−/–^ mice fed a WD was higher compared to those fed a CD (Figures 4A,B). However, irrespective of diet and gender, Aire deficiency did not influence plaque growth.

## Discussion

We herein demonstrate that deficiency of the TF AIRE, which regulates expression of many TRAs, does not affect generation and activation of CD4^+^ T cells responding to the ApoB peptide p6 in *Apoe*^−/–^ mice exposed to a WD. Irrespective of Aire, ApoB^+^ T cells exhibited a mixed proinflammatory T_H_1 T_H_17 T_FH_ phenotype, which is in line with previous data (5). AIRE deficiency associated with a reduction in thymic T_regs_ in female *Apoe*^−/–^ mice and with a slight decrease in the frequency of lymph node ApoB^+^ T_regs_ (which was only significant when compared with male but not female *Aire*^+/+^ mice). Given that no relevant alterations of other lymph node ApoB^+^ T cell subtypes could be detected in *Aire*^−/−^ mice, the influence of AIRE on phenotypes of ApoB^+^ T cells was overall modest and inconsistent. Alterations in the frequencies of T_regs_ or T_H_1 cells have been shown to substantially impact atherogenesis: Depletion of T_regs_ induced a 1.5–2 fold increase in lesion size (6, 7), whereas expansion of T_regs_ through active immunization strategies reduced atherosclerosis by more than 50% in mice (32). Likewise, genetic or antibody-mediated depletion of T_H_1 cells conferred significant atheroprotection (33, 34). However, in the present study, AIRE-deficiency influenced atherogenesis neither in CD- nor in WD-fed *Apoe*^−/–^ mice. We thus conclude that the minor phenotypic alterations of ApoB^+^ T cells in Aire-deficient mice do not reflect a clinically relevant effect. Rather, our findings collectively suggest that AIRE does not regulate thymic expression of ApoB peptides or other autoantigens triggering proatherogenic adaptive immune responses. Conflicting findings whether AIRE affects thymic ApoB expression have been reported (15, 24, 25). ApoB is a large protein and these inconsistencies might be attributable to the technology and sequences used to detect ApoB (microarray vs. bulk transcriptomics) (15, 24). It can thus not be excluded that thymic expression of some ApoB peptides might be AIRE-dependent. Yet, we show here that the CD4^+^ T cell response toward ApoB p6 and atherosclerotic burden was unaffected by AIRE-deficiency.

Our study has several limitations. We analyzed atherosclerotic plaque are exclusively in the aorta and not in the aortic root. As aortic atherosclerotic plaque area was unchanged by Aire-deficiency in all groups, we also refrained from extensive phenotyping of the atherosclerotic plaque environment for cellular composition and structural alterations. We thus cannot exclude, that Aire-deficiency might affect these parameters. It should be noted, that we only analyzed CD4^+^ T cells responding to the ApoB peptide, p6, which limits the generalizability of our findings. The choice of the p6 peptide was motivated by recent studies, in which p6-reactive CD4^+^ T cells were conclusively identified as important modulators of atherogenesis (5, 35, 36). The restriction to p6 does not allow direct inferences on the role of Aire in modulating generation, activation, or phenotypic alterations of CD4^+^ T cells responding to other ApoB peptides. However, a clinically relevant effect can almost certainly be excluded, since Aire-deficiency had absolutely no influences on atherogenesis.

Whereas, ApoB has been shown to be expressed by mTECs (23–25), we herein observed no impact of AIRE on atherogenesis and on ApoB-reactive CD4 T cells. We thus suggest that other mediators of central tolerance might be involved in modulating proatherogenic immune responses. Besides AIRE, FEZF2 was identified to regulate TRA gene expression in mTECs (16). Additionally, DCs and B cells are implicated in thymic presentation of circulating self-antigens and negative selection of autoreactive T cells (12). Recent evidence suggested that peripheral mechanisms might play a dominant role in mediating proatherogenic immunity. Particularly, polyclonal and, more importantly, ApoB^+^ T_regs_ undergo a phenotypic transition into proatherogenic T_H_1/T_reg_, T_FH_ (37, 38), or T_H_1/T_H_17 subtypes (5). Such phenotype switching also applied to adoptively transferred ApoB^+^-T_regs_ that failed to confer atheroprotection to *Apoe*^−/–^ mice (5). Adaptive immunity against ApoB is centrally involved in atherogenesis and specific therapies to modulate this proatherogenic immune responses do not yet exist. Hence, it is of high clinical relevance to clarify mechanisms of central and/or peripheral immune tolerance to ApoB. Particularly, the roles of FEZF2, thymic DCs and B cells in enabling central tolerance to ApoB and the underlying mechanisms of ApoB T cell phenotype switching need to explored in future studies.

Women are much more susceptible to several autoimmune diseases than men (39). AIRE expression was shown to be induced by androgen (40) but attenuated by estrogen (41), which might contribute to gender differences in prevalence of autoimmune diseases. Herein, we only detected minor and overall inconsistent immunological differences between male and female mice, whereas the overall phenotype regarding atherosclerotic lesion size and immunological parameters did not differ. Thymic generation of bulk T_regs_ was modestly increased in female compared to male *Aire*^+/+^*Apoe*^−/−^ mice. Considering the role of Aire in inducing thymic generation of some T_regs_ (28, 29) and the above-mentioned modulation of *Aire* expression by sex hormones, an increased T_reg_ frequency in the thymi of male compared to female *Aire*^+/+^ mice would have been expected. Why the opposite was the case in our study, remains unclear and warrants exploration in future studies. Secondly, in comparison to male *Aire*^+/+^*Apoe*^−/−^ mice, females harbored increased CD4^+^, CD8^+^, and γδ T cell counts in the lymph nodes. Additionally, some inconsistent variations of blood monocytes, neutrophils and lymphocytes between Aire-competent males and females were observed, which exclusively appeared either in CD- or WD-fed mice. Increased CD4^+^ T cells frequencies have been reported in female *Apoe*^−/–^ mice (42) and humans (43). Yet, contrary to our findings, previous studies detected higher numbers of CD8^+^ T cells in male *Apoe*^−/–^ mice and humans compared to females and women, respectively (42, 43). We recently reported on increased IL-17, IFNg, and IL-10 production in p6-ApoB^+^ CD4^+^ T cells in comparison to ApoB-negative CD4^+^ T cells (5). We here focused on the analysis of CD4^+^ T cell lineage transcription factors in p6-reactive CD4^+^ T cells, which was largely unaffected by Aire-deficiency. We do not report on the production of intracellular cytokines in p6-reactive CD4^+^ T cells and cannot exclude any effects that Aire-deficiency cause in these cells. Bulk CD4^+^ T cells of female mice exhibited increased IL-4 production. Also women exhibit a predominant T_H_2 cytokine profile compared to men (44), which may be explained by elevated IL-4 production in progesterone-treated CD4^+^ T cells (45). It may thus be interesting to investigate whether IL-4 harbors specific atheroprotective properties in females. Further work will be needed to elucidate the confirmability and biological importance of herein-revealed sex-related differences in immune cell composition during atherogenesis.

In conclusion, this study excludes AIRE as a mediator of central immune tolerance to ApoB and player in atherogenesis. Clarification of mechanisms underlying proatherogenic auto-immunity represents a fundamental requirement for development of anti-atherosclerotic immune therapies, which are currently lacking.

## Data Availability Statement

The original contributions presented in the study are included in the article/Supplementary Material, further inquiries can be directed to the corresponding author/s.

## Ethics Statement

All animal experiments were conducted in accordance with the institutional guideline for the La Jolla Institute for Immunology animal facility.

## Author Contributions

FN and HW wrote the manuscript, prepared the figures, and analyzed the data. FN, SB, KK, MO, MV, JM, RS, AA, DW, and HW performed experiments. HW and KL conceptualized and supervised the work and provided funding. All authors substantially contributed to data research, critically discussed the content, reviewed the manuscript before submission, and read and agreed to the published version of the manuscript.

## Funding

This research was funded by the Deutsche Forschungsgemeinschaft SFB TRR259 (397484323) and CCRC GRK2407 (360043781 to HW), the Neven-DuMont foundation to HW, the Koeln Fortune Program (363/2020 to FN), and HL115232, HL88093, and HL121697 from the National Heart, Lung and Blood Institute to KL.

## Conflict of Interest

The authors declare that the research was conducted in the absence of any commercial or financial relationships that could be construed as a potential conflict of interest.

## Publisher's Note

All claims expressed in this article are solely those of the authors and do not necessarily represent those of their affiliated organizations, or those of the publisher, the editors and the reviewers. Any product that may be evaluated in this article, or claim that may be made by its manufacturer, is not guaranteed or endorsed by the publisher.
