# Supplementary material for: Autoimmune Regulator (AIRE) Deficiency Does Not Affect Atherosclerosis and CD4 T Cell Immune Tolerance to Apolipoprotein B
Source: Front Cardiovasc Med. 2022 Jan 13;8:812769. doi: 10.3389/fcvm.2021.812769 (PMC8792778; doi:10.3389/fcvm.2021.812769)
Supplement: Supplementary file 1 [file Data_Sheet_1.PDF]

Supplementary Material

Supplementary Table 1. Body weights and hematological parameters.

	Aire$^{+/+}$ Apoe$^{-/-}$ F	*Aire$^{-/-}$ Apoe$^{-/-}$ F*	*Aire$^{+/+}$ Apoe$^{-/-}$ M*	*Aire$^{-/-}$ Apoe$^{-/-}$ M*
	**fed western-type diet for 12 weeks**			
n	12	10	10	16
Body weight (g)	25 ± 3	27,4 ± 1,7	33,8 ± 2,1****,†††	33,9 ± 4,2****,††††
Leukocytes (10^3/µl)	10,7 ± 4,4	18,1 ± 10,9	11 ± 4,7	13,2 ± 7,1
Neutrophils (10^3/µl)	3,9 ± 1,9	8 ± 5*	4,6 ± 2,4	5,9 ± 3,4
Lymphocytes (10^3/µl)	5,5 ± 2,7	8 ± 4,9	5,2 ± 2,2	5,6 ± 3,3
Monocytes (10^3/µl)	0,7 ± 0,3	1,1 ± 0,7	0,6 ± 0,2†	0,6 ± 0,4
Eosonophils (10^3/µl)	0,4 ± 0,3	0,8 ± 0,4	0,5 ± 0,4	0,9 ± 0,8
Basophils (10^3/µl)	0,1 ± 0,1	0,3 ± 0,1	0,2 ± 0,2	0,2 ± 0,2
Neutrophils (%)	36 ± 10,1	43,4 ± 5,3	39,9 ± 11	45 ± 12,2
Lymphocytes (%)	51,8 ± 10,4	44,5 ± 5,7	50,3 ± 12,1	43,1 ± 10
Monocytes (%)	7,1 ± 2,1	6 ± 0,8	5 ± 1,1*	4,7 ± 1,6**
Eosinophils (%)	4 ± 2,4	4,6 ± 1,6	3,7 ± 2,2	5,8 ± 3,7
Basophils (%)	1,1 ± 0,9	1,6 ± 0,8	1 ± 0,9	1,3 ± 1
Erythrocytes (10^6/µl)	8,8 ± 3,3	10,3 ± 1	9,8 ± 0,8	9,6 ± 3,2
Hemoglobin (g/dl)	12,7 ± 4,8	14,6 ± 1,6	13,4 ± 1,6	13,8 ± 4,8
HCT (%)	46,6 ± 16,9	54,8 ± 5,8	51,8 ± 5,5	51,5 ± 17,3
MCV (fl)	53,2 ± 2,4	53,4 ± 2,7	52,6 ± 1,5	53,3 ± 2,1
MCH (pg)	14,4 ± 0,7	14,3 ± 1,1	13,6 ± 0,9	14,3 ± 1
MCHC (g/dl)	27,1 ± 1,4	31,1 ± 14,1	25,8 ± 1,7	26,8 ± 1,4
RDW (%)	19,9 ± 2,3	22 ± 5,4	17,8 ± 3,2†	18,8 ± 1,4
Platelets (10^3/µl)	423 ± 291	475 ± 250	795 ± 400	746 ± 379
MPV (fl)	5,2 ± 0,4	5,3 ± 0,3	5,5 ± 0,3	5,2 ± 0,3
	**fed chow diet**			
n	12	16	8	3
Body weight (g)	24,4 ± 2	24,6 ± 2,1	30,4 ± 2,1****,††††	31 ± 1,4****,††††
Leukocytes (10^3/µl)	7,6 ± 2,3	11,7 ± 6,4	9,4 ± 4,8	10,8 ± 1,8
Neutrophils (10^3/µl)	2,2 ± 0,7	4,1 ± 2,5	4,1 ± 1,2	3,9 ± 1,3
Lymphocytes (10^3/µl)	4,8 ± 1,6	6,2 ± 2,7	5,5 ± 3,1	4,9 ± 0,1
Monocytes (10^3/µl)	0,4 ± 0,3	0,9 ± 0,8	0,6 ± 0,4	0,8 ± 0,1
Eosonophils (10^3/µl)	0,1 ± 0,1	0,4 ± 0,5	0,2 ± 0,2	1 ± 0,6**,‡‡
Basophils (10^3/µl)	0 ± 0	0,1 ± 0,1	0 ± 0,1	0,1 ± 0,1
Neutrophils (%)	29,7 ± 6,7	33,6 ± 5,9	41,3 ± 13,5*	35,6 ± 6,2
Lymphocytes (%)	62,9 ± 8,1	56,4 ± 8,3	51,3 ± 10,8*	46,1 ± 7,3*
Monocytes (%)	5,7 ± 2,7	6,7 ± 2,7	5,3 ± 2,5	7,9 ± 1,7
Eosinophils (%)	1,3 ± 1,1	2,8 ± 2,2	1,7 ± 1,8	9,1 ± 4,4****,†††,‡‡‡‡
Basophils (%)	0,3 ± 0,4	0,6 ± 0,7	0,4 ± 0,4	1,3 ± 0,5*
Erythrocytes (10^6/µl)	10,2 ± 0,8	9,6 ± 1	9,7 ± 0,7	10,4 ± 0,4
Hemoglobin (g/dl)	14,5 ± 1	14,1 ± 1,5	14,8 ± 1,4	15,7 ± 0,6
HCT (%)	53,5 ± 5,2	50,2 ± 4,6	52 ± 3,2	56,5 ± 2,6
MCV (fl)	52,5 ± 4,7	52,3 ± 3,8	53,8 ± 3,3	54,5 ± 0,6
MCH (pg)	14,2 ± 0,4	14,6 ± 0,8	15,5 ± 2,4	15,1 ± 0,2
MCHC (g/dl)	27,2 ± 2,1	28,1 ± 2,1	28,8 ± 4,3	27,8 ± 0,6
RDW (%)	18,1 ± 0,8	18,4 ± 0,9	18,6 ± 1,6	17,5 ± 0,4
Platelets (10^3/µl)	1038 ± 221	873 ± 362	1264 ± 656	386 ± 129‡
MPV (fl)	4,9 ± 0,4	5,1 ± 0,3	4,8 ± 0,4	4,9 ± 0,2

Statistical significance was determined by one-way ANOVA with Tukey's multiple comparisons test. * $p < 0.05$, ** $p < 0.01$, **** $p < 0.0001$ vs. Aire$^{+/+}$ F, † $p < 0.05$, ††† $p < 0.001$, †††† $p < 0.0001$ vs. Aire$^{-/-}$ F, ‡ $p < 0.05$, ‡‡ $p < 0.01$, ‡‡‡‡ $p < 0.0001$ vs. Aire$^{+/+}$ M

Supplementary Figures

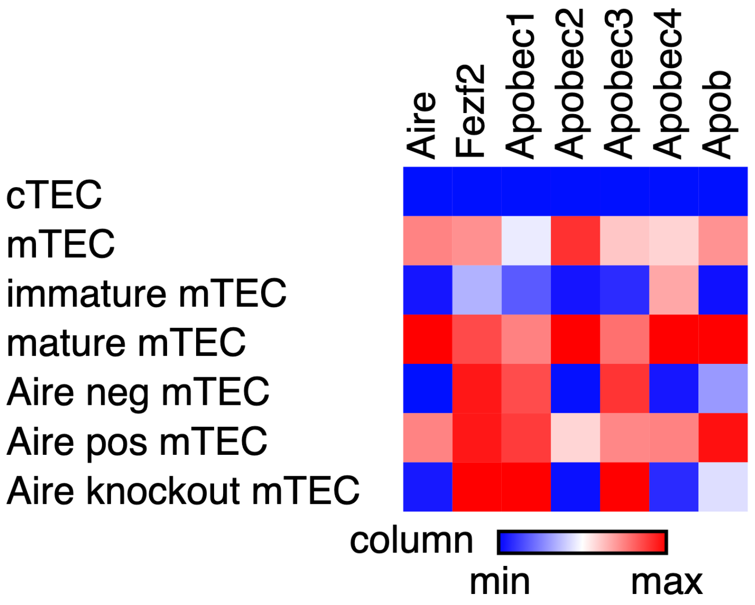

Supplementary Figure 1. Expression of transcription factors Aire, Fezf2; ApolipoproteinB (Apob) mRNA editing enzymes (Apobec) 1-4; and Apob in different thymic cell types. The heatmap shows column z-score-normalized Cuffdiff FPKM (Fragments Per Kilobase Million) values. Data was derived from (GEO accession GSE53111) (1).

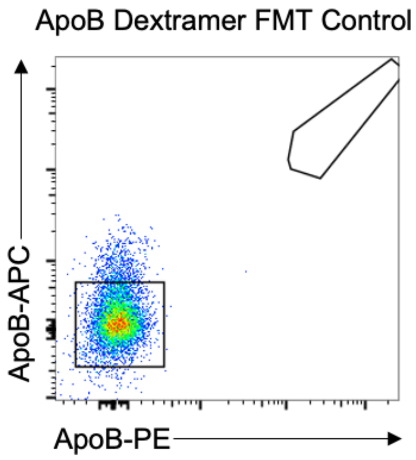

Supplementary Figure 2. Fluorescence-minus-two (FMT) control (PE and APC) of the ApoB dextramer gated on CD4+ T cells.

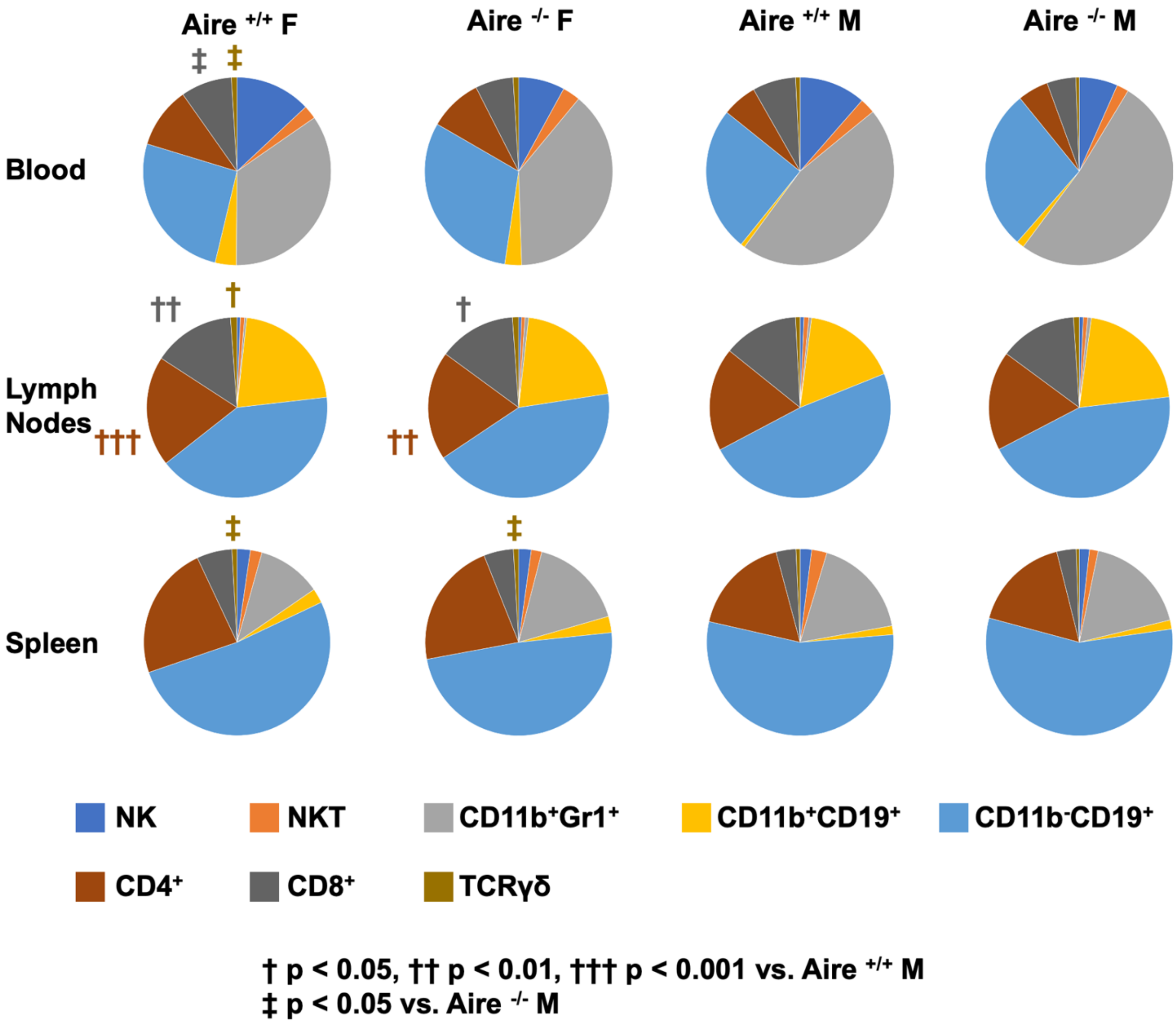

Supplementary Figure 3. Leukocyte composition in the blood, lymph nodes, and spleen. Natural killer (NK) cells, Natural killer T (NKT) cells, $Ly6G^+$ neutrophils and inflammatory $Ly6C^{high}$ monocytes ($CD11b^+Gr1^+$ cells), $CD11b^+CD19^+$ B1-like B cells, $CD11b^-$ $CD19^+$ B2 cells, $CD4^+$, $CD8^+$ and γδ T cells were quantified by flow cytometry in 20-week-old male and female *Aire*$^{-/-}$*Apoe*$^{-/-}$ and *Aire*$^{+/+}$*Apoe*$^{-/-}$ fed western-type diet for 12 weeks. n = 8-9 per group. Pie charts show the group means of indicated cell types. Statistical significance was determined by one-way ANOVA with Tukey's multiple comparisons test.

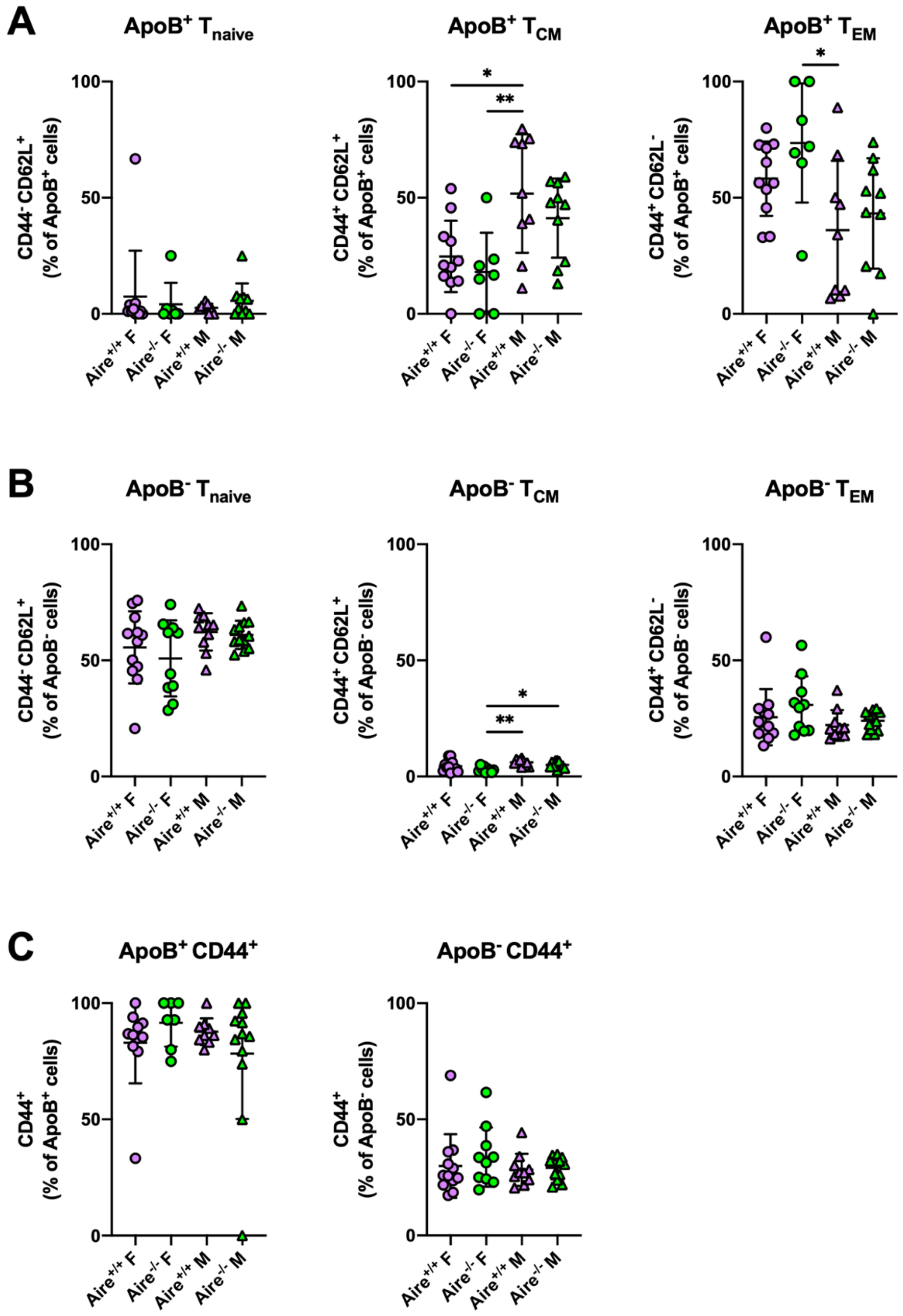

Supplementary Figure 4. Activation of ApoB$^+$ and ApoB$^-$ cells is not influenced by Aire deficiency. Quantification of naïve, effector and central memory (**A**) ApoB$^+$ and (**B**) ApoB$^-$ CD4$^+$ T cells isolated from lymph nodes of 20-week-old male and female *Aire*$^{-/-}$*Apoe*$^{-/-}$ and *Aire*$^{-+/+}$*Apoe*$^{-/-}$ fed western-type diet for 12 weeks. (**C**) Frequency of activated (all CD44$^+$) ApoB$^+$ and ApoB$^-$ cells. (**A, B**) Scatter plots correspond to the pie charts displayed in Figure 2C. (**A-C**) n = 7-11 per group. Data are expressed as

mean ± SD. Statistical significance was determined by one-way ANOVA with Tukey's multiple comparisons test. * $p < 0.05$, ** $p < 0.01$.

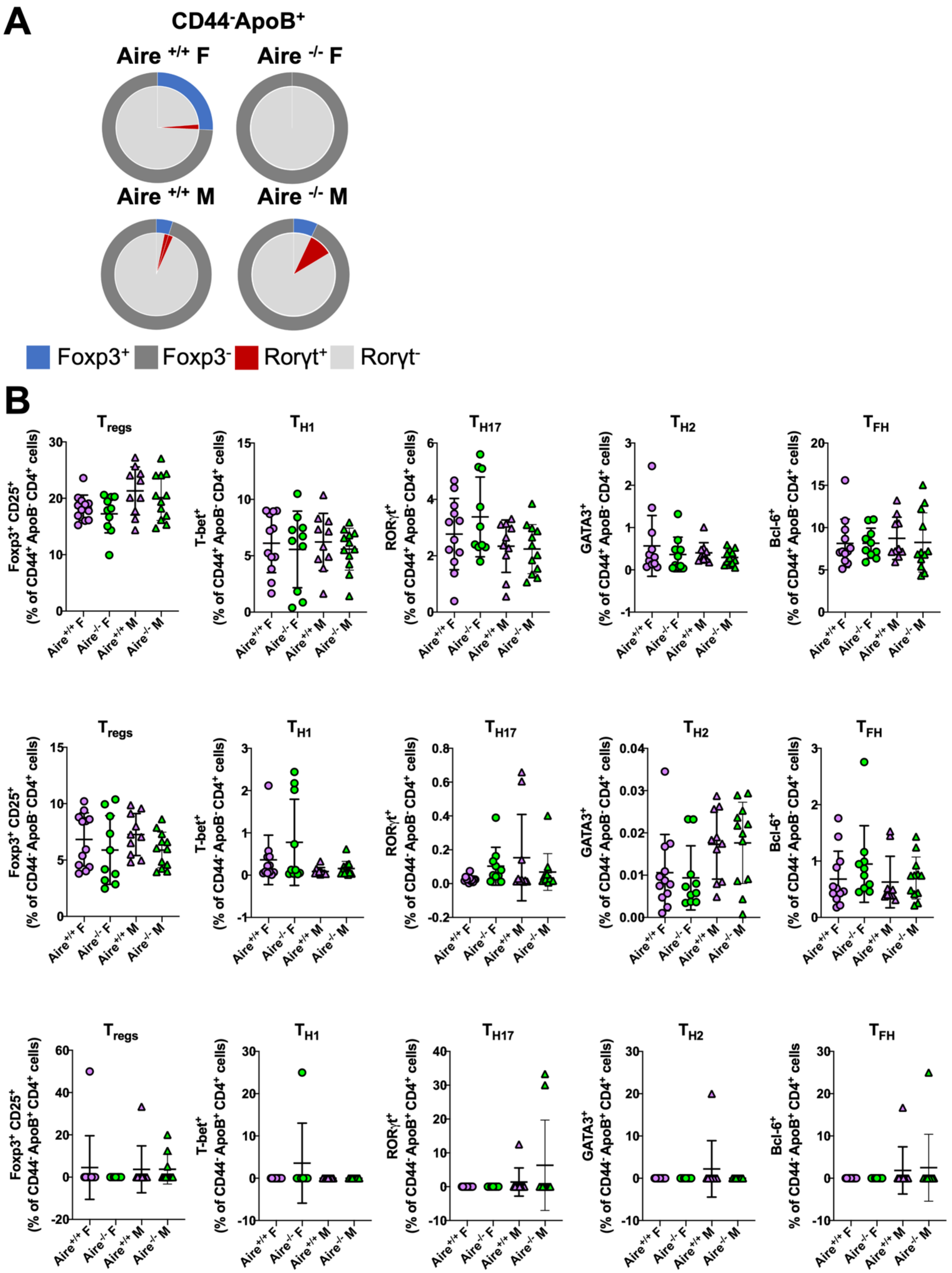

Supplementary Figure 5. Phenotypes of ApoB$^-$ and CD44$^-$ ApoB$^+$ cells are not affected by Aire deficiency. (**A**) Quantification of Foxp3$^+$ and RORγt$^+$ cells among CD44$^-$ ApoB$^+$ cells isolated from lymph nodes of 20-week-old male and female *Aire*$^{-/-}$*Apoe*$^{-/-}$ and *Aire*$^{+/+}$*Apoe*$^{-/-}$ fed western-type diet

for 12 weeks. Pie charts show the group means of indicated cell types. (**B**) Quantification of CD4 T cell lineage transcription factors T-bet (T_H1), GATA3 (T_H2), BCL6 (T_{FH}), Foxp3 (T_{regs}) and RORγt (T_H17) among CD44$^+$ApoB$^-$, CD44$^-$ApoB$^-$, and CD44$^-$ApoB$^+$cells. Data are expressed as mean ± SD. (**A and B**) n = 7-11 per group. Statistical significance was determined by one-way ANOVA with Tukey's multiple comparisons test.

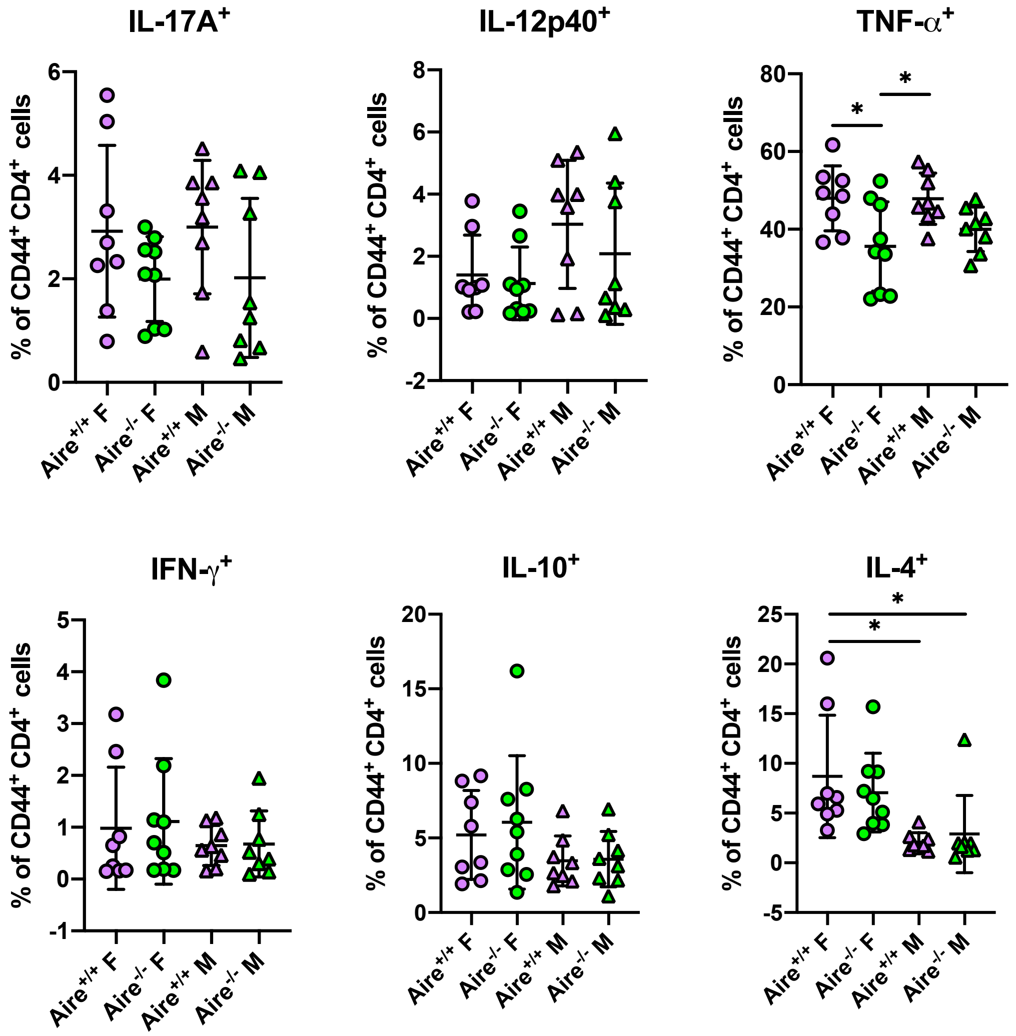

Supplementary Figure 6. Intracellular cytokine staining of $CD44^+$ $CD4^+$ T cells from pooled lymph nodes. Data are expressed as mean ± SD. n = 8-9 per group. Statistical significance was determined by one-way ANOVA with Tukey's multiple comparisons test.

References

1. Sansom SN, Shikama-Dorn N, Zhanybekova S, Nusspaumer G, Macaulay IC, Deadman ME, Heger A, Ponting CP, Holländer GA. Population and single-cell genomics reveal the Aire dependency, relief from Polycomb silencing, and distribution of self-antigen expression in thymic epithelia. *Genome Res* (2014) **24**:1918–1931. doi:10.1101/GR.171645.113
